# Child and adolescent psychiatry in the post-COVID era: lessons learned and consequences for the future

**DOI:** 10.1007/s00787-023-02208-6

**Published:** 2023-04-24

**Authors:** Michael Kaess, Pieter J. Hoekstra

**Affiliations:** 1 University Hospital of Child and Adolescent Psychiatry and Psychotherapy, University of Bern, Bern, Switzerland; 2 Department of Child and Adolescent Psychiatry, Centre for Psychosocial Medicine, University Hospital Heidelberg, Heidelberg, Germany; 3 Department of Child and Adolescent Psychiatry and Accare Child Study Center, University of Groningen, University Medical Center Groningen, Groningen, Netherlands

Since its beginning in early 2020, the COVID-19 pandemic has been one of the most challenging global health crises in modern times. While the acute phase of the pandemic seems to have run out after 3 years, and most societies worldwide have gotten back to daily businesses, a time-delayed “youth mental health crisis” is ongoing and currently one of the major challenges of many healthcare systems worldwide. This current crisis is marked by a dramatic increase in numbers of young individuals who seek professional help in the mental healthcare system (e.g. [1]). Given the already tight resources allocated to child and adolescent mental health before the pandemic, this increase currently leads to crowded and overwhelmed emergency units alongside with huge waiting lists for regular assessments and interventions.

Besides the current consequences for the clinical work in child and adolescent psychiatry, the COVID-19 pandemic could probably be seen as the largest naturalistic experiment that ever imposed a stressor to whole humankind and required a worldwide response of all healthcare systems. From a scientific point of view, there appear two major areas in that the pandemic—besides its dramatic and negative consequences—could at least be used to learn and draw beneficial consequences for the future of child and adolescent psychiatry. First, the COVID-19 pandemic came with the potential to investigate the effects of the pandemic as a major social stressor on the development of mental illness, including its pathways and mechanisms. This experiment of nature also allows for the identification of risk and protective factors for its mental health consequences. Second, the pandemic has given us the opportunity to learn to improve the delivery of our clinical practice. The required immediate response of our healthcare systems sharply illustrated the limitations of child and adolescent psychiatry services and pointed to important needs of innovations and solutions that may serve the future of our profession.

In this special topic section, we collected articles on what researchers found out on the two areas above, researchers who were able to quickly implement data collection and ask important research questions in midst of the chaotic world of the COVID-19 pandemic. Before we get to summarize some of the content of this focused issue of European Child and Adolescent Psychiatry, we would like to take the opportunity to thank all professionals (nurses, physicians, psychotherapists, scientists alike, or anybody else) in the field of child and adolescent mental health for their passionate and outstanding performance during the years of the pandemic. We would like to honor all of those who stood and still stand up for the rights and needs of our youngest, those who tried and still try to provide the best possible care under difficult circumstances, and those who currently try to improve our knowledge and our healthcare to improve the mental health of our next generation!

Getting back to the topic that the pandemic enabled us to gain insights on how a global stressor may affect child and adolescent mental health, this issue includes a variety of interesting articles that demonstrate potentially conflicting results of the effect of the pandemic. A systematic review in this issue provides increasing evidence for a longitudinal deterioration in the mental health of adolescents and young people, with increased levels of depression, anxiety, and psychological distress after the pandemic started [2]. This finding was indicated by several original articles in this issue [3–5]; however, some studies also included in this issue yielded no or a questionable overall impact of the pandemic [6, 7]. Conflicting evidence on the overall impact has previously been reported (e.g. divergent results from two population-based studies in Germany; [8, 9]), but comparisons of studies are often limited by differing methods of sampling, assessment, and analyses. Besides the methodological limitations, it is likely that the pandemic has not affected all individuals in the same way and that conflicting results may derive from different individual trajectories of mental health during the pandemic. In this issue, several risk and protective factors that may have influenced development of mental health in response to the pandemic are reported. An important risk factor for a deterioration was belonging to a vulnerable group, e.g. those with pre-existing trauma, pre-existing mental health problems, lower socioeconomic status or financial difficulties, poor family functioning or low social support [3, 4, 10]. More exciting, some individuals seemed to show resilience to or even benefit from the pandemic if they reported reduced bullying, more sleep, and/or more exercise [7].

The immediate response that the COVID-19 pandemic required led to increased demands for psychosocial interventions under circumstances of reduced options for face-to-face consultations. While this was a massive challenge for all of us, the potential of online-support for young people has increased during the pandemic [11], which has certainly stimulated the use of new technologies and communication tools within the field of child and adolescent psychiatry. Most clinicians worldwide were suddenly forced to conduct diagnostic assessments and therapeutic interventions via telemedicine. In this issue, researchers also report results from implementations of new ways to provide digitalized care to young individuals, e.g. via online peer support or serious gaming smartphone tools [12, 13]. We expect that the stimulus of the pandemic will results in major advances in the field of E-mental health, a field that was already on the rise before. However, as also described in this issue, this will require adaptation of guidelines and training for child and adolescent psychiatrists to facilitate the adequate use of telemedicine as well as new digital tools for diagnosis and treatment [14, 15].

We have all gone through a very challenging albeit interesting time and the consequences of the pandemic for child and adolescent psychiatry are still ongoing. As with every crisis, there are lessons to learn from, and it is now our job to draw the right consequences for the future. Given that this is likely not the last mental health crisis, a major consequence for the future should include the development of mental healthcare systems that are more flexible in terms of both capacities and treatment setting, which can likely be achieved by appropriate resources blended with new technologies.
